# Differential Glucocorticoid-Dependent Regulation and Function of the *ERRFI1* Gene in Triple-Negative Breast Cancer

**DOI:** 10.1210/endocr/bqaa082

**Published:** 2020-05-20

**Authors:** Chromewell Agustin R Mojica, Weand S Ybañez, Kevin Christian V Olarte, Alyssa Beatrice C Poblete, Pia D Bagamasbad

**Affiliations:** National Institute of Molecular Biology and Biotechnology, University of the Philippines Diliman, Quezon City, Philippines

**Keywords:** glucocorticoid, triple-negative breast cancer, ERRFI1, gene regulation

## Abstract

Glucocorticoids (GCs; eg, hydrocortisone [CORT]) are routinely used as chemotherapeutic, anti-emetic, and palliative agents in breast cancer (BCa) therapy. The effects of GC signaling on BCa progression, however, remain a contentious topic as GC treatment seems to be beneficial for receptor-positive subtypes but elicits unfavorable responses in triple-negative BCa (TNBC). The mechanistic basis for these conflicting effects of GC in BCa is poorly understood. In this study, we sought to decipher the molecular mechanisms that govern the GC-dependent induction of the tumor suppressor *ERRFI1* gene, an inhibitor of epidermal growth factor receptor (EGFR) signaling, and characterize the role of the GC-*ERRFI1* regulatory axis in TNBC. Treatment of TNBC cell lines with a protein synthesis inhibitor or GC receptor (GR) antagonist followed by gene expression analysis suggests that *ERRFI1* is a direct GR target. Using in silico analysis coupled with enhancer-reporter assays, we identified a putative *ERRFI1* enhancer that supports CORT-dependent transactivation. In orthogonal assays for cell proliferation, survival, migration, and apoptosis, CORT mostly facilitated an oncogenic phenotype regardless of malignancy status. Lentiviral knockdown and overexpression of *ERRFI1* showed that the CORT-enhanced oncogenic phenotype is restricted by ERRFI1 in the normal breast epithelial model MCF10A and to a lesser degree in the metastatic TNBC line MDA-MB-468. Conversely, ERRFI1 conferred pro-tumorigenic effects in the highly metastatic TNBC model MDA-MB-231. Taken together, our findings suggest that the progressive loss of the GC-dependent regulation and anti-tumorigenic function of ERRFI1 influences BCa progression and may contribute to the unfavorable effects of GC therapy in TNBC.

Breast cancer (BCa) is the most prevalent type of malignancy worldwide and is the leading cause of cancer-related death in women (1). The molecular heterogeneity of BCa influences the clinical management of this disease, with diagnosis and treatment relying on the expression status of the relevant receptors: estrogen receptor (ER), progesterone receptor (PR), and human epidermal growth factor receptor 2 (HER2) (2,3). Immunohistochemical and gene expression analyses define the molecular subtype triple-negative BCa (TNBC) based on the lack of ER and PR expression, and HER2 amplification (4). The absence of these molecular targets renders TNBC difficult to treat, with therapeutic strategies limited to systemic approaches such as chemotherapy and radiation therapy (5,6). Patients with TNBC, thus, have worse prognosis with a higher risk of relapse compared with other BCa subtypes (7).

Included in the chemotherapeutic regimen for BCa is the administration of glucocorticoids (GCs), either as monotherapy or as adjuvants with cytotoxic agents (8,9). Glucocorticoids are steroid hormones that play central roles in animal development, and in physiological and behavioral responses to stress (10-12). At the cellular level, GCs exert their actions by binding to the GC receptor (GR), which functions as a ligand-activated transcription factor (13). Glucocorticoids initially gained prominence in the clinic as potent anti-inflammatory agents used to treat diseases caused by a hyperactive immune system (14). In cancer, GCs have been routinely employed in the clinical management of hematopoietic malignancies because of their ability to induce apoptosis (15). The effects of GC treatment in non-hematopoietic malignancies remain a controversial theme in clinical oncology (16,17). For instance, GCs are found to be beneficial in hormone-responsive BCa, as they are able to inhibit cell proliferation and suppress inflammatory response to chemotherapy and radiation (18). In the TNBC subtype, however, GC treatment becomes ineffective and may even confer therapeutic resistance (19). Our knowledge of the underlying mechanisms that dictate this paradoxical shift in the effectiveness of GCs remains fragmentary.

Transcriptome analyses of the normal mammary epithelial cell line MCF10A (20) and the TNBC model cell line MDA-MB-231 (21) treated with the synthetic GC dexamethasone (DEX) showed that GCs regulate genes associated with cell survival, cell invasion, and epithelial-to-mesenchymal transition (EMT). A GC-inducible gene that has been implicated in cell cycle control is the tumor suppressor *ErbB Receptor Feedback Inhibitor 1* (*ERRFI1*, also known as *MIG6* or *RALT*) (22,23) gene, which encodes for a scaffold adaptor protein that inhibits the activity of the ErbB receptor family, including the epidermal growth factor receptor (EGFR) (22,24). The ability of ERRFI1 to block EGFR signaling occurs by a dual mechanism where ERRFI1 directly binds to EGFR to inhibit EGFR catalytic activity (24) and targets EGFR for lysosomal degradation (25). *ERRFI1* is an immediate early response gene and its expression can be induced by a broad spectrum of stimuli such as growth factors, hormones, and stress (22,26-30). *ERRFI1* is an attractive subject to study in the context of GC-mediated effects in TNBC because although several studies have shown that the expression of *ERRFI1* is induced by GC treatment (23,30-33) and downregulated in BCa (34,35), the molecular basis of the regulatory action of GCs on *ERRFI1* gene expression remains to be resolved.

To improve our understanding of TNBC biology and expand the limited knowledge on the effects of GCs on BCa and the characteristic tumor-suppressive properties of ERRFI1, we sought to investigate the GC-*ERRFI1* regulatory axis in the context of TNBC. In this study, we demonstrate the regulatory logic that governs GC-dependent induction of *ERRFI1*, and the potential role that this axis plays in BCa progression and the GC therapy paradox. By combining gene expression analysis, in silico enhancer identification, and enhancer-reporter assays, we found that *ERRFI1* is a direct GR target, and we identified an 821-bp enhancer element located ~21.5 kb downstream of the *ERRFI1* transcription start site (TSS) that supports GC-dependent transactivation. In complementary cellular assays on cancer hallmarks, we found that ERRFI1 restricts the pro-tumorigenic effect of CORT in the normal breast epithelial model MCF10A and to a lesser degree in the metastatic TNBC line MDA-MB-468. In the highly metastatic TNBC model MDA-MB-231, ERRFI1 lost its tumor suppressive capacity and instead conferred pro-tumorigenic effects.

## Materials and Methods

### Cell culture

MCF10A (RRID:CVCL_0598) (36), MDA-MB-468 (RRID:CVCL_0419) (37), and MDA-MB-231 (RRID:CVCL_ 0062) (38) were obtained from Celina G. Kleer of the University of Michigan Medical School. MCF10A is a cell line commonly used to model normal mammary epithelia (39). MDA-MB-468 is metastatic BCa cell line with a distinct *EGFR* amplification (40) associated with poor clinical outcome (41,42). MDA-MB-231 is a breast metastatic cell line that expresses markers associated with EMT and stemness, and is used to model highly aggressive BCa (43). All cell lines do not express ER, PR, and HER2 (39,43) and were used as models for triple-negative breast epithelia in the study. In addition, the 3 cell lines express GR protein with MDA-MB-231 having the highest GR protein level, followed by MDA-MB-468, and MCF10A having the lowest GR level (44). Cell lines were authenticated by Macrogen (Korea) using short tandem repeat profiling (Powerplex 21 System, Promega), and tested negative for mycoplasma contamination using the Microsart AMP Mycoplasma Kit (Sartorius).

MCF10A cells were cultured in DMEM/F12 (Gibco, 12500-062) supplemented with 2.438 g/L sodium bicarbonate, 5% horse serum (HS; Gibco, 16050–114), 10 µg/mL insulin (Invitrogen, 12585-014), 100 ng/mL cholera toxin (Sigma, C8052), 1 µg/mL hydrocortisone (CORT; Sigma, H4001), 10 ng/mL EGF (Invitrogen, 02633), 1X penicillin-streptomycin (Gibco, 10378-016). MDA-MB-231 and MD-MB-468 were cultured in RPMI-1640 (Gibco, 31800-022) supplemented with 2.0 g/L sodium bicarbonate, 10% fetal bovine serum (FBS), 10 µg/mL insulin, and 1X penicillin-streptomycin. All cell lines were incubated in a humidified environment at 37°C and 5% CO2.

### Hormone treatment

For gene expression analysis, MCF10A (3.0 × 10^5^ cells/well), MDA-MB-231 (3.0 × 10^5^ cells/well), and MDA-MB-468 (4.0 × 10^5^ cells/well) were seeded into 12-well plates in complete media. When cells reached 70% confluency, cells were hormone-deprived by changing media into DMEM/F12 with 5% charcoal steroid stripped (CSS)-HS for MCF10A, and RPMI-1640 with 10% CSS-FBS for MDA-MB-231 and MDA-MB-468. Prior to hormone treatments, cells were starved in serum-free media overnight. CORT (Sigma, H0888) was dissolved in ethanol and added to wells at various concentrations (0.01087% final ethanol concentration) for 2 h before harvesting ribonucleic acid (RNA) for dose-response analysis. To determine kinetics of CORT-dependent *ERRFI1* induction, cells were treated with 100 nM CORT for 0.5, 1, 2, and 4 h before harvest for RNA extraction. Dose-response and time-course experiments were performed twice with comparable results.

To determine if *ERRFI1* is a direct GR target or if the CORT-dependent induction of *ERRFI1* requires ongoing protein synthesis, cells were pretreated with 100 µg/mL of the protein synthesis inhibitor cycloheximide (CHX; Sigma, 01810) in serum-free medium for 30 min before and during CORT (300 nM) treatment. To evaluate whether the effects of CORT treatment is mediated by and specific to GR, we pretreated cells with 1 µM of the GR-selective antagonist mifepristone (RU486; Sigma, M8046) in serum-free medium for 1 h before and during CORT (300 nM) treatment. Cycloheximide and RU486 were dissolved in 100% ethanol. All CORT treatments were continued for 2 h before harvest for RNA extraction. The CHX and RU486 experiments were performed at least twice with similar results.

### RNA extraction and gene expression analysis

We extracted total RNA using the TRIzol reagent (Invitrogen, 15596-018) following the manufacturer’s protocol. One microgram of total RNA was used for complementary deoxyribonucleic acid (cDNA) synthesis using the High Capacity Reverse Transcription kit with ribonuclease inhibitor (Applied Biosystems, 4374967). Gene expression analysis was performed by quantitative polymerase chain reaction (PCR) using PowerUp SYBR Green Master Mix (Applied Biosystems, A25742) run in a real-time PCR (ABI 7500 Fast). All primers (Table 1) used for measuring messenger ribonucleic acid (mRNA) expression spanned exon/intron boundaries and a relative quantification was done using a pool of cDNAs. For measuring precursor mRNA (pre-mRNA) levels, total RNA was treated with DNAse I (Sigma, AMPD1), primers (Table 1) for transcript expression targeted *ERRFI1* intron 1 and exon 2 sequences, and real-time quantitative PCR without reverse transcriptase did not yield amplicons. Melt curve analysis was also completed to determine specificity of amplification by primers. We normalized transcript levels to the expression levels of the reference gene *18s rRNA*, which were unaffected by hormone treatments and did not vary between cell lines.

**Table 1.** Primers used for SYBR green-based quantitative real-time PCR analysis for gene expression

Target	Forward	Reverse
*18s rRNA*	5’-GGATGTAAAGG ATGGAAAATACA-3’	5’-TCCAGGTCTTC ACGGAGCTTGTT-3’
*KLF9* mRNA	5’-GAGCAGTCGCA GTGAGTTTA-3’	5’-TCATCGGAGCA GATTTGGAAG-3’
*ERRFI1* mRNA	5’- CTGGAGCAGT CGCAGTGAG-3’	5’- GCCATTCATCGGAGCAGATTTG -3’
*ERRFI1* pre-mRNA	5’- CTCTCGTTCA TTCCAGGGC-3’	5’-AGCTGGACTT TTGAGATGGAC-3’
m*Errfi1* mRNA	5’-CATCTCGAGTGAG GCAGTACACAGGAAG-3’	5’-GCGAAGCTTGTG AAAGGCAGAATGAGTC-3’
*ID2* mRNA	5’-ATCCCACTAT TGTCAGCCTGC-3’	5’-TGAACACCGC TTATTCAGCCAC-3’
*ID2* pre-mRNA	5’-CCTCTGCCCT TAGGTTACATT-3’	5’-AAAGAAATC ATGAACTGC-3’
*EGFR* mRNA	5’-CAAGGAAGCCA AGCCAAATG-3’	5’-CCGTGGTCAT GCTCCAATAA-3’
*NR3C1* mRNA	5’-CCGTGGTCAT GCTCCAATAA-3’	5’-CACCTTCCTGTC TCCTGTTTAC-3’

### Identification and validation of the *ERRFI1* downstream enhancer

To identify candidate enhancer regions, we analyzed publicly available GR chromatin immunoprecipitation-sequencing (ChIP-seq) data on MCF10A (GSE102355) (45) and MDA-MB-231 (GSE56022) (21) cells treated with DEX (100 nM) for 1 h. ENCODE data (46) on active chromatin marks such as RNA polymerase II (Pol II) binding (GSE94062) (45), H3K27 hyperacetylation (H3K27Ac) sites, DNase I hypersensitivity peaks, and conservation across vertebrate species were also assessed through the UCSC genome browser based on Human Feb. 2009 (GRCh37/hg19) (47). We further refined our in silico search by evaluating predicted long-range interactions in the GeneHancer database (48) and conservation of GC response elements (GREs) through the transcription factor binding site search algorithm and visualization webtool LASAGNA 2.0 (49).

Using genomic DNA extracted from MCF10A cells, PCR primers (Table 2) were designed to amplify an 821-bp DNA fragment corresponding to the predicted enhancer located ~21.5 kb downstream of the *ERRFI1* TSS. The *ERRFI1* downstream enhancer (EDE) was subcloned into the pGL4.23[luc2/minP] luciferase vector (Promega, E8411) at the SacI and HindIII sites to generate plasmids for enhancer-reporter assays.

**Table 2.** Primers used for generating pGL4.23-*ERRFI1* downstream enhancer reporter and pLKO.1-sh*ERRFI1* construct

Target	Forward	Reverse
EDE	5’-CATGAGCTCTGGTATT GAGGTGACGCATTAG-3’	5’-GTCAAGCTTGGAACCG CAAGCAAGTAAAC-3’
sh*ERRFI1*	5’-CCGGCTAGACCAGTAAA GCCAGATTCTCGAGAATCGGCTTTACTGGTCTAGTTTTTG-3’	5’- AATTCAAAAACTAGACC AGTAAAGCCAGATTCTCGAGAATCTGGCTTTACTGGTCTAG-3’

Abbreviation: EDE, ERRFI1 downstream enhancer.

For the dual luciferase reporter assays, MCF10A and MDA-MB-468 cells were seeded in 24-well plates at 1.0 × 10^5^ and 2.5 × 10^5^ cells per well, respectively. At 70% confluency, cells were hormone-starved by changing media into DMEM/F12 with 5% CSS-HS for MCF10A and RPMI-1640 with 10% CSS-FBS for MDA-MB-468. Cells were then transfected with 475 ng of pGL4.23-EDE or pGL4.23- upstream mouse *Cyb561* enhancer (UCE) as a positive control (50), and 25 ng of the normalization reporter p*Renilla* luciferase-thymidine kinase construct (Promega, E2241) using the XtremeGENE HP DNA Transfection Reagent (Roche, 6366236001) following the manufacturer’s protocol. Immediately before transfection, the growth medium was replaced with medium containing CSS, and cells were incubated with the transfection mixture overnight. Following overnight incubation with the transfection complex, cells were pre-treated with vehicle or 1 µM RU486 for 1 h in corresponding medium with CSS. Cells were then treated with vehicle or CORT (300 nM) for 20 h before harvest for luminescence assays using the Dual-Luciferase Reporter Assay System (Promega, E1980) and the FluoroskanTM FL Microplate Luminometer (ThermoScientific). Enhancer-reporter assays were done twice with 3 to 4 replicates per treatment.

### Lentiviral-mediated knockdown and overexpression of *ERRFI1*

The short hairpin RNA (shRNA) construct targeting *ERRFI1* (sh*ERRFI1*; TRCN0000118131; Genetic Perturbation Platform shRNA library, Broad Institute) and scrambled shRNA pLKO.1 (RRID:Addgene_1864) (51) were used for lentiviral production. The sh*ERRFI1* construct was generated by ligating the annealed shRNA oligos (Table 2) into the pLKO.1 vector (RRID:Addgene_8453) (52) digested with AgeI and EcoRI. Viral particles were packaged in HEK293T cells by transfecting the pLKO.1-sh*ERRFI1* or pLKO.1-scrambled shRNA constructs with viral packaging plasmids (pHCMVG, pRSV-Rev, pMDLg/pRRE) using the Effectene Transfection Reagent (Qiagen, 301425). Media with viral particles was collected twice (48 and 72 h post-transfection) and filtered through 0.45 µm polyethersulfone membrane. MCF10A, MDA-MB-231, and MDA-MB-468 cells were transduced by media with viral particles supplemented with 8 µg Polybrene transfection reagent (Merck, TR-1003-G). Transduced cells were then selected using complete media with 2.0 µg/mL puromycin (Gibco, A11138-03).

The mouse homolog of the human *ERRFI1* gene (81.6% homology) with 100% conserved EGFR-interacting domains was used in overexpression experiments. The *mErrfi1* gene was subcloned into the AsiSI and MluI sites of the pLenti-C-Myc-DDK (OriGene, PS100092) construct. Lentiviral particles were generated as previously described. Validation of knockdown and overexpression was done by real-time quantitative PCR.

### Colony formation assay

MCF10A (1.5 × 10^3^ cells/well), MDA-MB-231 (1.5 × 10^3^ cells/well), and MDA-MB-468 (3.0 × 10^2^ cells/well) knockdown or overexpression lines were seeded into 6-well plates in complete media. Twenty-four hours after seeding, media was then changed to DMEM/F12 with 2.5% CSS-HS or RPMI with 2.5% CSS-FBS and incubated overnight. The cells were then treated with vehicle or CORT (100 nM; 0.0036% final ethanol concentration). Hormone was replenished every third day, and each treatment was done in triplicate. After 14 days, colonies were fixed with methanol-acetic acid (3:1) solution and stained with 0.5% crystal violet. Wells were scanned using HP Deskjet Ink Advantage 4645. A cell colony is defined as a group of more than 50 cells. Colonies were manually counted using the Vision SX45 Stereomicroscope. Experiments were repeated twice with similar results.

### CyQuant direct cell proliferation assay

MCF10A (3.0 × 10^3^ cells/well), MDA-MB-231 (2.0 × 10^3^ cells/well), and MDA-MB-468 (3.0 × 10^3^ cells/well) scrambled shRNA- and sh*ERRFI1*-transduced cells were seeded into 96-well clear-bottom black plates (Falcon, 353219) in complete media. Twenty-four hours after seeding, cells were starved in DMEM/F12 with 5% CSS-HS or RPMI with 10% CSS-FBS overnight. The cells were then treated with vehicle or CORT (100 nM; 0.0036% final ethanol concentration). Cell proliferation was measured at the 0 and 72 h hormone treatment timepoint by incubating with CyQuant Reagent (Invitrogen, C35011) for 1 h. Bottom-read fluorescence was then measured with excitation wavelength of 480 nM and emission of 535 nM using EnSight Multimode Plate Reader (PerkinElmer). Each treatment had 5 replicates for each timepoint, and all experiments were repeated twice with similar results.

### Trypan blue exclusion assay

MCF10A (1.0 × 10^4^ cells/well), MDA-MB-231 (7.5 × 10^3^ cells/well), and MDA-MB-468 (1.0 × 10^4^ cells/well) scrambled shRNA- and sh*ERRFI1*-transduced cells were seeded into 6-well plates in complete media. Twenty-four hours after seeding, cells were starved in DMEM/F12 with 5% CSS-HS or RPMI with 10% CSS-FBS overnight. The cells were then treated with vehicle or CORT (100 nM; 0.0036% final ethanol concentration). Hormone was replenished every three days after initial treatment. On the fifth day from the initial treatment, cells in each well were harvested by trypsinization. The cells were then quantified by diluting the cell suspension with trypan blue (Gibco, 15250-061) at 1:1 ratio prior to manual cell count using a hemocytometer, with the experimenter performing the count blind to treatment. Each treatment was done in triplicate and experiments were repeated twice with similar results.

### PrestoBlue cell viability assay

MCF10A (3.0 × 10^3^ cells/well), MDA-MB-231 (2.0 × 10^3^ cells/well), and MDA-MB-468 (3.0 × 10^3^ cells/well) knockdown and overexpression lines were seeded into 96-well clear-bottom black plates (Falcon, 353219) in complete media. Twenty-four hours after seeding, cells were starved in DMEM/F12 with 5% CSS-HS or RPMI with 10% CSS-FBS overnight. The *ERRFI1* knockdown cell lines were then treated with vehicle or CORT (100 nM; 0.0036% final ethanol concentration) for 24, 48, 72, and 96 h. For *mErrfi1* overexpressing lines, an endpoint assay was performed with fluorescence measured at the 0 and 72 h timepoints. Hormone was replenished every 24 h until the 96 h timepoint. Cell viability for every timepoint was measured after incubation with PrestoBlue (Invitrogen, A13262) for 30 mins. Bottom-read fluorescence was then measured with excitation wavelength of 560 nM and emission of 590 nM using EnSight Multimode Plate Reader (PerkinElmer). Each treatment had 5 replicates for each timepoint, and all experiments were repeated at least twice with similar results.

### Wound-healing assay

MDA-MB-468 and MDA-MB-231 scrambled shRNA- and sh*ERRFI1*-transduced cells were seeded into 12-well plates at a density of 4.0 × 10^5^ and 2.0 × 10^5^ cells/well, respectively. When the cells reached confluency, the cells were starved in 1% CSS-FBS overnight. To minimize possible confounding effects of cell proliferation, the cells were pre-incubated in 10 µg/mL mitomycin C (Millipore, 475820) for 2 h prior to the scratch. Thereafter, a wound was made by dragging a P200 tip to create an open gap. Cell debris was then removed by washing with PBS twice. The cells were then treated with vehicle or CORT (500 nM; 0.018% final ethanol concentration) added to RPMI-1640 with 1% CSS-FBS for MDA-MB-468 or RPMI-1640 with 0.5% CSS-FBS for MDA-MB-231. Immediately after treatment, wound closure was monitored by taking images of the wound at 0 h timepoint (baseline) and after 24 h of hormone treatment using the Olympus IX51 Inverted Microscope. Wound area was analyzed using the MRI Wound Tool Macro in the ImageJ software (53). Percentage wound closure was designated as the average of the area difference of the two images taken in each well relative to the baseline. Each treatment had 5 replicates and experiments were performed twice with similar results.

### CellEvent Caspase-3/7 apoptosis assay

MDA-MB-231 (8.0 × 10^3^ cells/well) and MDA-MB-468 (1.0 × 10^4^ cells/well) scrambled shRNA- and sh*ERRFI1*-transduced cells were seeded into 96-well clear-bottom black plates (Falcon, 353219) in complete media. When the cells reached 80% confluency, they were hormone-deprived by incubation with RPMI + 10% CS-FBS overnight. The cells were then treated with CORT (100 nM) and doxorubicin (DOX; 5 μM or MDA-MB-23; 0.5 μM for MDA-MB-468; Sigma, D1515) (54). After 48 h, apoptosis was measured by labeling cells with 5 μM CellEvent Caspase-3/7 Green Detection Reagent (Invitrogen, C10423) for 30 min. Bottom-read fluorescence was then measured with excitation wavelength of 502 nM and emission of 530 nM using EnSight Multimode Plate Reader (PerkinElmer). Fluorescently-labeled cells were imaged using the Olympus IX51 Inverted Microscope. Each treatment had 4 replicates and experiments were performed twice with similar results.

### Statistical analysis

Data for gene expression analysis (normalized to *18s rRNA* transcript levels), dual luciferase assays (Firefly luciferase counts divided by *Renilla* luciferase counts), and cell proliferation and viability (fluorescence signal at timepoint divided by signal at 0 h) were log_10_ transformed before statistical analysis. The dose-response, time-course, RU486 gene expression, and dual luciferase assay data were analyzed using 1-way analysis of variance (ANOVA) followed by Tukey’s post-hoc test, while data from the CHX assay were analyzed using the Student’s unpaired *t*-test. Data from the colony formation, cell proliferation, and cell viability assays were analyzed using 2-way ANOVA to test for main effects of CORT treatment and *ERRFI1* expression, followed by Student’s unpaired *t*-test to determine effects of CORT within a shRNA or overexpression line and effect of *ERRFI1* expression between same hormone treatment. Apoptosis assay data were analyzed using 2-way ANOVA followed by Tukey’s post-hoc test to assess interaction of CORT treatment and *ERRFI1* expression. All statistical analyses were done using GraphPad Prism version 8.0 (GraphPad Software, La Jolla, CA, US, www.graphpad.com), and *P* < 0.05 was accepted as statistically significant.

## Results

### CORT induces *ERRFI1* mRNA in a dose- and time-dependent manner

**Figure 1.**
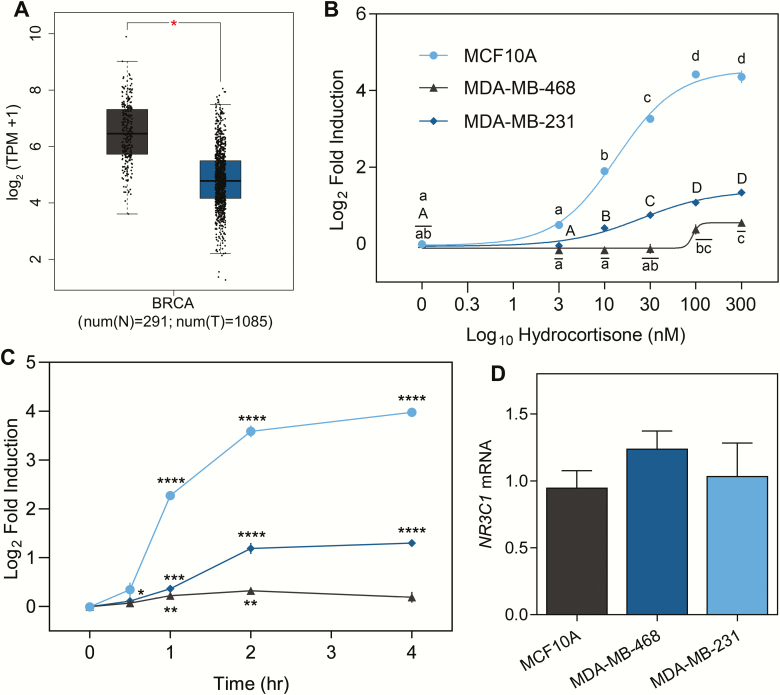
*ERRFI1* is downregulated in breast cancer tumor samples and is differentially induced by CORT in TNBC. **(A)** Genotype-Tissue Expression Project (GTEx) data for normal breast tissue (56) and the Cancer Genome Atlas breast invasive carcinoma RNA-seq data (from the Cancer Genome Atlas Research Network: http://www.cancer.gov/tcga) (57) were analyzed using the online tool Gene Expression Profiling Interactive Analysis (55). *ERRFI1* is significantly downregulated in breast cancer samples compared to normal tissue equivalents (*P* < 0.01). **(B)** MCF10A, MDA-MB-468, and MDA-MB-231 cells (n= 3-4/treatment) were treated with increasing doses of CORT for 2 h. In MCF10A cells, dose-dependent increase in *ERRFI1* mRNA expression levels was observed upon CORT treatment (1-way ANOVA; F(5,17) = 573.4; *P* < 0.0001). In MDA-MB-468 cells, a significant increase in *ERRFI1* mRNA was observed starting at 100 nM CORT and did not change with increasing CORT dose (1-way ANOVA; F(5,17) = 11.55; *P* < 0.0001). In MDA-MB-231, *ERRFI1* mRNA levels increased with increasing CORT dose (1-way ANOVA; F(5,18) = 63.67; *P* < 0.0001) but with a lower magnitude of induction compared to MCF10A cells. **(C)** All 3 TNBC lines were treated with 100 nM CORT for the timepoints indicated before harvest and real-time quantitative PCR analysis. In MCF10A and MDA-MB-468, a significant increase in *ERRFI1* expression level relative to the vehicle-treated control was observed after 1 h (Student’s *t*-test; MCF10A, *P* < 0.0001; MDA-MB-468, *P* = 0.01) while in MDA-MB-231, *ERRFI1* mRNA expression significantly increased after 30 mins (Student’s *t*-test; MDA-MB-231: *P* = 0.0179). **(D)** All 3 TNBC lines expressed similar levels of *NR3C1* (*GR*) mRNA (1-way ANOVA; F(2,8) = 0.9548; *P* = 0.4248). *ERRFI1* mRNA levels were normalized to the reference gene *18s rRNA*, which was unaffected by hormone treatment. The normalized values were log_10_ transformed prior to statistical analysis. Dose-response curves were fitted by nonlinear regression. Dots represent the log_2_(fold induction) ± standard error of the mean while bars represent mean ± standard error of the mean with statistical significance indicated by asterisks in Student’s *t*-test (*P < 0.05, **P < 0.01, ***P < 0.001, ****P < 0.0001) or letters above the means (lowercase for MCF10A, uppercase for MDA-MB-231, and lowercase with an overline for MDA-MB-468) in 1-way ANOVA (means with the same letter are not significantly different; Tukey’s multiple comparison test; *P* < 0.05). Experiments were performed twice with consistent results and graphs shown are representative of the different trials.

Expression studies have shown that *ERRFI1* is downregulated in several human tumor types (35) and BCa cell lines (34). Using the Gene Expression Profiling Interactive Analysis (GEPIA) tool (55), we analyzed expression data derived from the Genotype-Tissue Expression Project (56) and the Cancer Genome Atlas breast invasive carcinoma RNA-seq data (57), and found that *ERFFI1* is significantly downregulated in BCa samples compared to normal tissue equivalents (Fig. 1A). To gain mechanistic insight on how GCs influence *ERRFI1* expression in BCa, we measured *ERRFI1* transcript levels in BCa cells in response to increasing doses of CORT (0-300 nM) for 2 h. In MCF10A cells, increasing CORT concentration led to a dose-dependent increase in *ERRFI1* mRNA levels (EC_50_ of 13.52 nM) with submaximal induction observed at 10 nM CORT (Fig. 1B). A dose-dependent response (EC_50_ of 25.34 nM) was also observed in MDA-MB-231 (Fig. 1B) but at a lower fold induction compared to MCF10A. In MDA-MB-468 cells, significant induction of *ERRFI1* mRNA was seen only with 100 nM CORT (Fig. 1B). In addition, the induction level (1.5-fold) observed with 300 nM CORT treatment of MDA-MB-468 cells is the lowest among the BCa cell line models. Treatment with 100 nM CORT also caused a time-dependent increase in *ERRFI1* transcript levels in MCF10A and MDA-MB-231, but not in MDA-MB-468 (Fig. 1C). In MCF10A, an increase in *ERRFI1* mRNA levels (4.83-fold) was initially observed at the 1-h timepoint and continued to increase through 4 h. In MDA-MB-231, *ERRFI1* mRNA levels increased slightly at 30 min (1.08-fold), peaked at 2 h (2.11-fold), and was sustained until 4 h of CORT treatment (Fig. 1C). The 3 cell lines had similar *GR* (*NR3C1*) transcript levels (Fig. 1D).

### *ERRFI1* is directly regulated by liganded-GR

**Figure 2.**
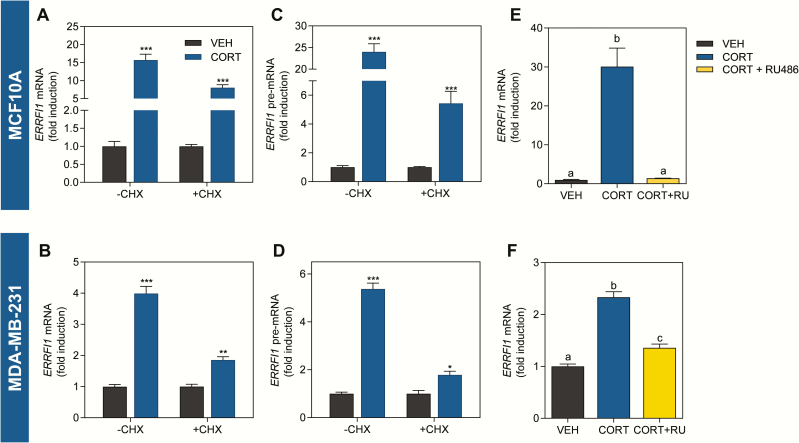
Induction of *ERRFI1* mRNA is resistant to protein synthesis inhibition and is abolished in the presence of a selective-GR antagonist. **(A)** MCF10A cells were incubated with 100 µg/mL CHX for 30 min before addition of CORT (300 nM). Treatment with CORT plus CHX was continued for 2 h before cell harvest for analysis of *ERRFI1* mRNA. Treatment with CORT significantly induced *ERRFI1* mRNA expression and CORT-dependent induction persisted in the presence of CHX (Student’s *t*-test; −CHX, *P* = 0.0001; +CHX, *P* < 0.0001). **(B)** CORT treatment of MDA-MB-231 cells significantly increased *ERRFI1* mRNA expression and is resistant to protein synthesis inhibition (Student’s *t*-test; −CHX, *P* < 0.0001; +CHX, *P* = 0.0006). CORT treatment likewise caused a statistically significant increase in *ERRFI1* pre-mRNA levels in **(C)** MCF10A (Student’s *t*-test; −CHX, *P* < 0.0001; +CHX, *P* < 0.0001) and in **(D)** MDA-MB-231 cells (Student’s *t*-test; −CHX, *P* < 0.0001; +CHX, *P* = 0.0099) that persisted with CHX treatment. Pre-incubation with 1 µM of the GR-selective antagonist mifepristone (MIF; RU486) for 1 h before addition of vehicle or CORT (100 nM) for 2 h abolished the CORT-dependent induction of *ERRFI1* mRNA in (E) MCF10A (1-way ANOVA; F(2,9) = 268.8, *P* < 0.0001) and (F) MDA-MB-231 (1-way ANOVA; F(2,9) = 78.92, *P* < 0.0001). *ERRFI1* mRNA levels were normalized to the *18s rRNA* housekeeping gene whose expression was not affected by hormone treatment, and normalized values were log_10_ transformed before statistical analysis. Bars represent the fold induction ± standard error of the mean relative to vehicle control with statistical significance indicated by asterisks in Student’s *t*-test (*P < 0.01, **P < 0.001, ***P < 0.0001) or letters above the means in 1-way ANOVA (means with the same letter are not significantly different; Tukey’s multiple comparison test; *P* < 0.05). All treatments were done with 3 to 4 replicates. Experiments were performed at least twice with consistent results. Graphs shown are representative of the different trials.

To determine if *ERRFI1* is a direct GR target, we treated MCF10A and MDA-MB-231 cells with the protein synthesis inhibitor CHX before and during CORT exposure. In both cell lines, the CORT-dependent increase in *ERRFI1* mRNA persisted even in the presence of CHX (Fig. 2A and B), similar to that of the known direct GR target *KLF9* (Supplemental Fig. 1A and B) (58,59). To distinguish whether the CORT-dependent induction observed after the 2-h treatment occurs via an increase in transcriptional rate or through increase in mRNA stability, we also measured the levels of the pre-mRNA. Consistent with *ERRFI1* mRNA, CORT treatment led to an increase in *ERRFI1* pre-mRNA that was also resistant to protein synthesis inhibition (Fig. 2C and D). In contrast, the CORT-dependent repression of the known indirect GR target gene *ID2* (60) was abolished upon the addition of CHX at both mRNA and pre-mRNA level (Supplemental Fig. 1C and D) (59) demonstrating that CHX effectively inhibited protein synthesis. To determine whether the CORT-dependent induction of *ERRFI1* mRNA is mediated by and specific to GR, we pre-treated MCF10A (Fig. 2E) and MDA-MB-231 (Fig. 2F) cells with the GR-selective antagonist RU486, which abolished the CORT-dependent induction of *ERRFI1* mRNA, confirming that the transcriptional regulation is mediated by GR.

### In silico analysis identifies a candidate *ERRFI1* enhancer that supports CORT-dependent transactivation

**Figure 3.**
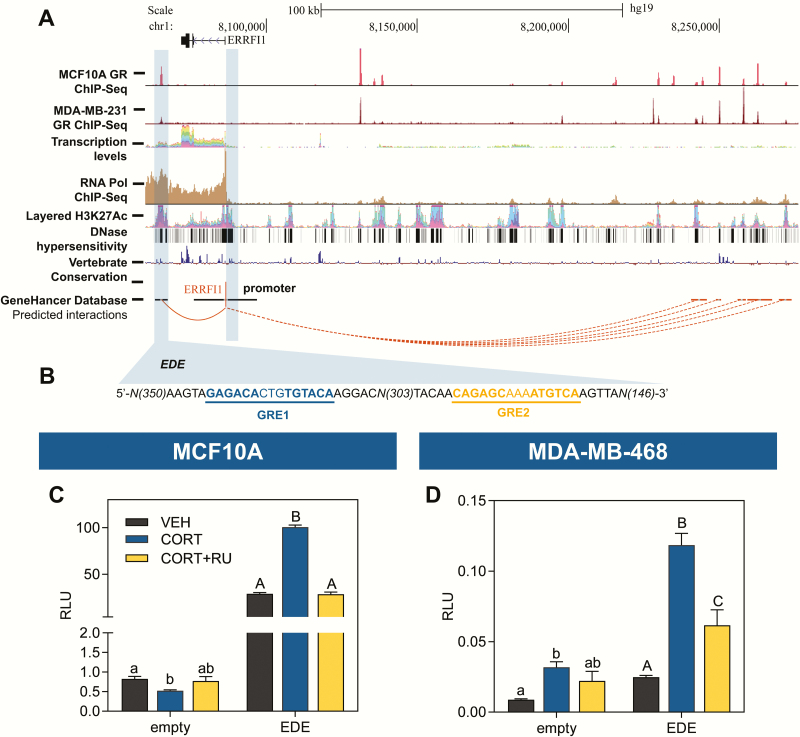
Identification of an 821-bp enhancer element downstream of the *ERRFI1* TSS that supports GR-mediated transactivation. **(A)** The UCSC Genome Browser (47) with annotation based on the human February 2009 (GRCh37/hg19) genome assembly was used to visualize the human *ERRFI1* locus and surrounding nongenomic regions. Highlighted are the candidate *ERRFI1* downstream enhancer (EDE) region located ~21.5 kb downstream of the *ERRFI1* TSS and the proximal promoter region. **(B)** The EDE contains 2 GREs predicted by LASAGNA 2.0 (49). MDA-MB-231 GR- (GSE56022) (21), and MCF10A GR- (GSE102355) and RNA Pol II ChIP-seq data (GSE94062) (45) were obtained from the Gene Expression Omnibus. The putative enhancer is a transcription factor binding hotspot, which is suggestive of a regulatory function and is enriched for active enhancer marks as demonstrated by analysis of ENCODE (46) data for noncoding transcripts based on RNA-seq, H3K27Ac ChIP-seq peaks, DNase I hypersensitivity clusters, vertebrate conservation, and predicted long-range interactions from the GeneHancer database (48). Enhancer luciferase constructs containing the 821-bp EDE or empty vector control were transfected into **(C)** MCF10A and **(D)** MDA-MB-468 cells. Twenty hours after transfection, cells were treated with vehicle (100% ethanol), CORT (300 nM), or RU486 (1 uM) plus CORT for 20 h before harvest and analysis by dual luciferase assay. In MCF10A and MDA-MB-468 cells, the EDE showed robust CORT-dependent transactivation that was abolished with RU486 treatment (1-way ANOVA; MCF10A: F(2,9) = 180.1, *P* < 0.0001; MDA-MB-468: F(2,9) = 24.72, *P* = 0.0002). The empty vector control showed slight CORT-induced transactivation that was not affected by RU486 (1-way ANOVA; MCF10A: F(2,9) = 6.075, *P* = 0.0214; MDA-MB-468: F(2,7) = 4.560, *P* = 0.0540). Relative luminescence units were obtained from Firefly luminescence normalized to the *Renilla* control whose activity did not change across treatments. Normalized values were log_10_ transformed before statistical analysis. Bars represent mean ± standard error of the mean and the letters above the mean indicate significant differences among treatments (means with the same letter are not significantly different; *P* < 0.05, Tukey’s multiple comparison test). All treatments were done with 3 to 4 biological replicates and all experiments were performed twice with consistent results.

To identify candidate CORT-responsive *cis*-regulatory elements in the *ERRFI1* locus, we used the following chromatin signatures representative of enhancer elements (61): (i) must have a GR ChIP-Seq peak based on GC-treated MCF10A (45) and MDA-MB-231 (21) data sets; (ii) has marks of an open chromatin environment such as RNA Pol II binding (45), H3K27 hyperacetylation, and DNase I hypersensitivity (46,47); (iii) must be conserved among vertebrates (46,47); and (iv) predicted to exhibit long-range interactions with the *ERRFI1* proximal promoter using the using the GeneHancer database (48). In silico analysis identified an 821-bp putative enhancer region located ~21.5 kb (chr1:8064775-8065595; +21 589 kb from the TSS) downstream of the *ERRFI1* TSS. The candidate EDE exhibited high GR localization in both MCF10A (GSE102355) (45) and MDA-MB-231 (GSE56022) (21) treated with 100 nM DEX for 1 h; displayed high RNA Pol II, H3K27Ac ChIP-seq peaks, and DNase I hypersensitivity sites; showed a high degree of sequence conservation among vertebrates; is predicted to interact with the *ERRFI1* promoter by chromosome looping (Fig. 3A); and contains 2 putative GREs (Fig. 3B). We tested the activity of the EDE in enhancer-luciferase reporter assays using MCF10A and MDA-MB-468 cells. Treatment of both cell lines with 300 nM CORT led to an increase in luciferase activity that was abolished in the presence of RU486 (Fig. 3C and D). The previously identified UCE (50) was used as a CORT-responsive positive control in MCF10A (Supplemental Fig. 2A) and MDA-MB-468 cells (Supplemental Fig. 2B) (59).

We did not find any appreciable GR peak in both GC-treated MCF10A and MDA-MB-231 GR ChIP-Seq data sets at regions surrounding the *ERFFI1* TSS including the proximal promoter up to 5 kb upstream the TSS (Fig. 3A and Supplemental Fig. 3 (59)). We also used LASAGNA to search for GREs from the *ERRFI1* TSS up to 5 kb upstream of the TSS and found only 1 candidate GRE that had a lower score than the GREs in the EDE (Table 3). More important, there was no GR ChIP peak at the corresponding GRE within the TSS up to 5 kb upstream of the *ERRFI1* TSS, in contrast to the GREs identified in the EDE, which had a corresponding GR ChIP peak.

**Table 3.** LASAGNA motif binding search of GRE in the EDE region and the proximal promoter region spanning 5kb upstream of the TSS

Name of TF model	Sequence	Strand	Position	Score	*P*-value	*E*-value
GR-alpha (T00337)	CAGAGCAAAATGTCA	+	20940 (EDE, GRE2)	130.9	0.000675	0.56
GR-alpha (T00337)	TGTACACAGTGTCTC	-	21268 (EDE, GRE1)	148.72	0.0001	0.082
GR-alpha (T00337)	GCATCACATTGACCC	+	-2846 (promoter)	129.49	0.00075	3.7

Sequences indicated are either in the +/- strand and positions are relative to the TSS set as the zero position. Scores are obtained for the GR-alpha transcription factor model from the TRANSFAC database (T00337) based on position-specific scoring matrices. The *P*-value is empirically computed from the position-specific scoring matrices scores of individual nucleotides in the sequence relative to the TF model (62) and is indicative of the probability of observing a score equal to or higher than the score by chance (63). The *E-*value considers the length of the genomic region being tested and gives the number of expected times a hit of the same or higher score is found in the genomic region by chance (63).

Abbreviation: TF, transcription factor.

### *ERRFI1* knockdown has different effects on oncogenic phenotype in TNBC

**Figure 4.**
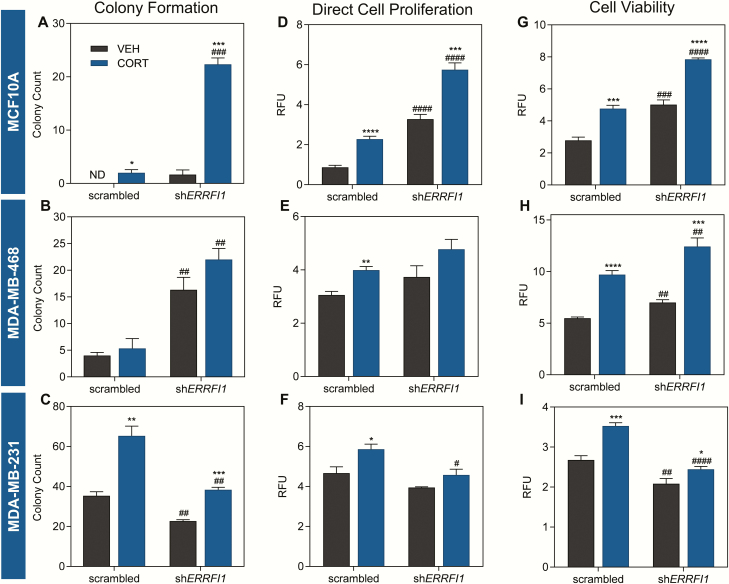
*ERRFI1* knockdown has different effects on cell survival, proliferation, and viability in TNBC. Effects of *ERRFI1* knockdown and CORT treatment on **(A-C)** cell survival, **(D-F)** direct cell proliferation, and (G-I) cell viability of TNBC cells were evaluated using assays based on colony formation, fluorescence-based DNA-binding, and resazurin reduction, respectively. For the colony formation assay, scrambled and sh*ERRFI1* cells were treated with vehicle (100% ethanol) or CORT (100 nM) for 14 days. For the direct cell proliferation and cell viability assay, cells were treated as previously described for 72 h. In MCF10A, CORT **(A)** enhanced colony formation (2-way ANOVA; Treatment factor: F(1,8) = 201.0, *P* < 0.0001; Knockdown factor: F(1,8) = 189.4, *P* < 0.0001), **(D)** increased cell proliferation (2-way ANOVA; Treatment factor: F(1,15) = 110.2, *P* < 0.0001; Knockdown factor: F(1,15) = 239.3, *P* < 0.0001), and (G) increased cell viability (2-way ANOVA; Treatment factor: F(1,14) = 97.85, *P* < 0.0001; Knockdown factor: F(1,14) = 118.2, *P* < 0.0001). These effects of CORT were enhanced by *ERRFI1* knockdown. CORT treatment of MDA-MB-468 cells **(B)** did not affect cell survival (2-way ANOVA; Treatment factor: F(1,8) = 3.615, *P* = 0.0938; Knockdown factor, F(1,8) = 62.04, *P* < 0.0001), **(E)** slightly augmented cell proliferation (2-way ANOVA; Treatment factor: F(1,12) = 13.38, *P* = 0.0033; Knockdown factor: F(1,12) = 6.215, *P* = 0.0283), (H) and increased cell viability (2-way ANOVA; Treatment factor: F(1,14) = 158.3, *P* < 0.0001; Knockdown factor: F(1,14) = 28.99, *P* < 0.0001). Knockdown of *ERRFI1* increased cell survival and viability independent of the pro-tumorigenic effects of CORT but had no effect on cell proliferation. In MDA-MB-231, CORT treatment **(C)** promoted colony formation (2-way ANOVA; Treatment factor: F(1,8) = 71.64, *P* < 0.0001; Knockdown factor: F(1,8) = 54.05, *P* < 0.0001), **(F)** enhanced proliferation (2-way ANOVA; Treatment factor: F(1,13) = 13.56, *P* = 0.0028; Knockdown factor: F(1,13) = 16.52, *P* = 0.0013), and **(I)** increased viability (2-way ANOVA; Treatment factor: F(1,16) = 31.27, *P* < 0.0001; Knockdown factor: F(1,16) = 61.37, *P* < 0.0001). Knockdown of *ERRFI1* conferred anti-tumorigenic effect in the cell line. For the cell proliferation and cell viability assays, measures were normalized to raw fluorescence reads at 0 h, which was set as baseline (relative fluorescence units), and normalized values were log_10_ transformed before statistical analysis. Bars represent mean ± standard error of the mean with statistical significance determined through Student’s *t-*test (*P < 0.05, **P < 0.01, ***P < 0.001, ****P < 0.0001 for statistically significant effects of CORT within a shRNA type, and ^#^*P* < 0.05, ^##^*P* < 0.01, ^###^*P* < 0.001, ^####^*P* < 0.0001 for statistically significant effects of *ERRFI1* knockdown between the same hormone treatment). For the colony formation assay, treatments were performed with 3 replicates, while for the direct cell proliferation and cell viability assays, treatments were done with 5 replicates. All experiments were performed at least twice with consistent results and graphs shown are representative of the different trials.

To characterize the role of the GC-*ERRFI1* regulatory axis in triple-negative breast epithelia, we stably expressed *ERRFI1*-specific shRNA and effectively knocked down *ERRFI1* expression by at least 50% in the model cell lines used in cellular assays (Supplemental Fig. 4) (59). In the colony formation assay using scrambled shRNA-transduced MCF10A cells, no colonies formed with vehicle, but treatment with CORT induced colony formation. This survival effect imparted by CORT was greatly enhanced by *ERRFI1* knockdown (22-fold increase in CORT-treated sh*ERRFI1* vs CORT-treated scrambled shRNA cells) (Fig. 4A and Supplemental Fig. 5A (59)). In metastatic MDA-MB-468 cells, CORT treatment did not affect the survival of scrambled shRNA-transduced cells. However, in vehicle-treated sh*ERRFI1* expressing cells, there was a 4.08-fold increase in the number of colonies compared to vehicle-treated scrambled shRNA-expressing cells. The effect of reduced *ERRFI1* expression on enhancing colony formation was not altered by CORT treatment (Fig. 4B and Supplemental Fig. 5B (59)). In the highly aggressive TNBC line MDA-MB-231 transduced with scrambled shRNA, CORT treatment led to a 1.9-fold increase in the number of colonies compared to vehicle treatment. This effect of CORT was dampened by *ERRFI1* knockdown (1.7-fold increase in CORT- vs vehicle-treated sh*ERRFI1*-transduced cells) (Fig. 4C and Supplemental Fig. 5C (59)). *ERRFI1* knockdown also led to an overall decrease in colony formation of MDA-MB-231 cells.

We next evaluated the role of the GC-*ERRFI1* axis on cell proliferation using a DNA-binding fluorescence-based proliferation assay. CORT treatment of scrambled shRNA transduced-MCF10A cells significantly increased cell proliferation (2.6-fold) (Fig. 4D and Supplemental Fig. 5D (59)). When comparing vehicle-treated cells, sh*ERRFI1*-transduced cells exhibited a significant increase in cell proliferation (3.76-fold increase) compared to scrambled shRNA cells. The effect of *ERRFI1* knockdown in increasing cell proliferation was further enhanced with CORT treatment (1.76-fold increase in CORT- vs vehicle-treated sh*ERRFI1* cells) (Fig. 4D). In scrambled shRNA-transduced MDA-MB-468 cells, CORT treatment increased cell proliferation, but this effect of CORT was not observed in sh*ERRFI1*-transduced cells (Fig. 4E and Supplemental Fig. 5E (59)). In scrambled shRNA-transduced MDA-MB-231 cells, CORT treatment caused a small but significant increase in cell proliferation (1.26-fold, *P* = 0.0187), and this effect of CORT was abolished with *ERRFI1* knockdown (Fig. 4F and Supplemental Fig. 5F (59)). In addition, we observed a decrease in cell proliferation when comparing CORT-treated sh*ERRFI1*-transduced MDA-MB-231 cells with CORT-treated scrambled shRNA-transduced cells. For all 3 cell lines, similar effects of CORT treatment and *ERRFI1* knockdown on cell proliferation were obtained using the trypan blue exclusion assay (Supplemental Fig. 5G-I) (59).

Since our DNA-binding based cell proliferation assay does not distinguish between live and dead cells, we performed an orthogonal resazurin-based assay to assess the effect of CORT treatment and *ERRFI1* knockdown on cell viability. In scrambled shRNA-transduced MCF10A cells, CORT treatment caused a 1.71-fold increase in cell viability compared to vehicle treatment and this effect of CORT was enhanced by *ERRFI1* knockdown (1.65-fold increase in CORT-treated sh*ERRFI1* vs CORT-treated scrambled shRNA-transduced cells) (Fig. 4G). When comparing vehicle-treated cells, there was also a 1.80-fold increase in cell viability of sh*ERRFI1* compared to scrambled shRNA-transduced MCF10A cells (Fig. 4G). CORT treatment of scrambled shRNA-transduced MDA-MB-468 cells increased cell viability by 1.77-fold compared to vehicle treatment, and this effect of CORT was enhanced upon *ERRFI1* knockdown (1.28-fold increase in CORT-treated sh*ERRFI1* vs CORT-treated scrambled shRNA-transduced cells) (Fig. 4H). In MDA-MB-231 scrambled shRNA cells, CORT treatment also increased cell viability by 1.32-fold, but this effect of CORT was diminished with *ERRFI1* knockdown (Fig. 4I). Moreover, we observed a decrease in cell viability when comparing vehicle or CORT-treated sh*ERRFI1* cells with their scrambled shRNA counterpart (0.26-fold decrease in cell viability of vehicle-treated sh*ERRFI1* compared to vehicle-treated scrambled shRNA-transduced cells; 0.44-fold decrease in cell viability of CORT-treated sh*ERRFI1* compared to CORT-treated scrambled shRNA-transduced cells) (Fig. 4I).

**Figure 5.**
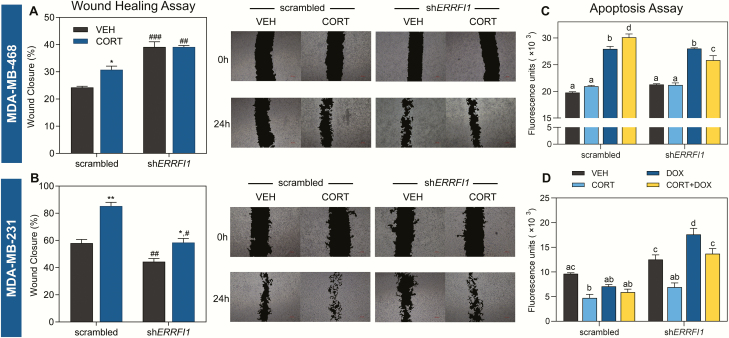
*ERRFI1* knockdown has different effects on cell migration and apoptosis of TNBC. Assessment of cell migration **(A and B)** was performed using the wound healing assay. Cells were treated with either vehicle (100% ethanol) or CORT (500 nM). Representative images of the scratch at each timepoint are shown to the right of each plot. **(A)** In MDA-MB-468, CORT treatment of scrambled shRNA-transduced cells increased migration but had no effect on sh*ERRFI1*-transduced cells. Knockdown of *ERRFI1*, however, increased cell migration compared to scrambled shRNA control cells with the same treatment (2-way ANOVA; Treatment factor: F(1,14) = 12.05, *P* = 0.0037; Knockdown factor: F(1,14) = 111.0, *P* < 0.0001). **(B)** In MDA-MB-231 cells, CORT treatment-enhanced cell migration of scrambled shRNA- and sh*ERRFI1*-transduced cells. However, *ERRFI1* knockdown decreased cell migration compared to scrambled shRNA control cells with the same hormone treatment (2-way ANOVA; Treatment factor: F(1,14) = 50.83, *P* < 0.0001; Knockdown factor: F(1,14) = 48.83, *P* < 0.0001). Apoptosis **(C and D)** of scrambled and *ERRFI1* knockdown cells was assessed using an assay based on the activity of caspase-3 and caspase-7 to cleave an amino acid peptide conjugated to a DNA-binding dye. Cells were treated with vehicle, CORT (100 nM), DOX (500 nM in MDA-MB-468; 5 µM in MDA-MB-231) and a combination of CORT plus DOX (n = 4/treatment) and apoptosis was measured 48 h after treatment. **(C)** In MDA-MB-468 cells, CORT enhanced DOX-induced apoptosis of scrambled shRNA-transduced cells. Upon *ERRFI1* knockdown, CORT had a protective effect against DOX-induced apoptosis (2-way ANOVA; Treatment factor: F(3,21) = 211.5, *P* < 0.0001; Knockdown factor: F(1,21) = 4.762, *P* = 0.0406). **(D)** In MDA-MB-231 cells, *ERRFI1* knockdown sensitized cells to the apoptotic effect of DOX, while CORT treatment suppressed the DOX-induced cytotoxicity (2-way ANOVA; Treatment factor: F(3,23) = 23.10, *P* < 0.0001; Knockdown factor: F(1,23) = 96.45, *P* < 0.0001). For the wound healing assay, percentage wound closure was designated as the average of the area difference of the 2 images taken in each well relative to the baseline measure at 0 h. These values were log_10_ transformed before statistical analysis. Bars represent mean ± standard error of the mean with statistical significance determined through Student’s *t-*test (*P < 0.01,**P < 0.001 for statistically significant effects of treatment within a shRNA type, and ^#^*P* < 0.01, ^##^*P* < 0.001, ^###^*P* < 0.0001 for statistically significant effects of *ERRFI1* knockdown between the same treatment) or through 2-way ANOVA followed by Tukey’s multiple comparisons test (bars with same letters above the means are not significantly different; *P* < 0.05). Treatments were performed with 4 to 5 replicates for both assays. All experiments were performed at least twice with consistent results and graphs shown are representative of the different trials.

The effects of CORT treatment and *ERRFI1* knockdown on cell migration of the TNBC cell lines MDA-MB-468 and MDA-MB-231 were also assessed through the scratch-wound assay. In MDA-MB-468 cells, CORT treatment of scrambled shRNA-transduced cells increased cell migration (Fig. 5A). Although this effect of CORT was lost in the sh*ERRFI1*-transduced line, cell migration increased in vehicle (14.86% increase) and CORT-treated (8.39% increase) sh*ERRFI1* lines compared to their scrambled shRNA equivalent (Fig. 5A). In MDA-MB-231 cells, CORT treatment of scrambled shRNA- and sh*ERRFI1*-transduced lines also increased cell migration, but the magnitude of CORT effect was reduced with *ERRFI1* knockdown (Fig. 5B). In addition, there was a decrease in wound closure of vehicle-treated (13.64% decrease) and CORT-treated (26.90% decrease) sh*ERRFI1* lines compared to their scrambled shRNA counterparts (Fig. 5B).

Finally, we evaluated the influence of the GC-*ERRFI1* axis on DOX-induced apoptosis in TNBC models using a fluorescence-based assay that measures caspase-3/7 activity. In MDA-MB-468 cells, *ERRFI1* knockdown did not affect DOX-induced cytotoxicity. In the scrambled shRNA-transduced cells, CORT enhanced the cytotoxic effects of DOX. This effect of CORT was reversed with *ERRFI1* knockdown such that CORT had a protective effect against DOX-induced cytotoxicity in sh*ERRFI1*-transduced MDA-MB-468 cells (Fig. 5C). In the aggressive MDA-MB-231 line, *ERRFI1* knockdown sensitized the cells to the cytotoxic effects of DOX. We also observed a protective effect of CORT against DOX-induced apoptotic effect when *ERRFI1* is knocked down (Fig. 5D).

### *Errfi1* overexpression decreased tumorigenicity of MDA-MB-468 cells

**Figure 6.**
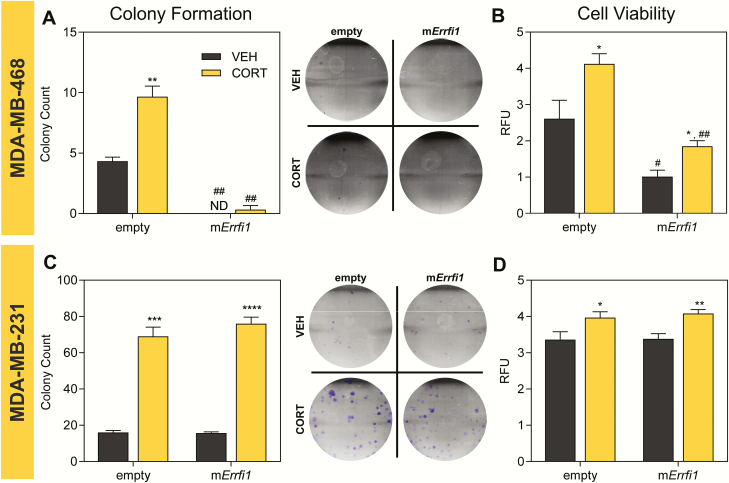
Overexpression of m*Errfi1* differentially alters cell survival and viability of TNBC. Effects of CORT treatment and m*Errfi1* overexpression on cell survival, and cell viability of MDA-MB-468 and MDA-MB-231 cells. Clonogenic assay **(A and C)** on empty or m*Errfi1* overexpressing cells (n = 3/treatment) treated with either vehicle or CORT (100 nM) for 14 days. Representative images of colonies stained with crystal violet are shown to the right of each plot. Cell viability (n = 4-5/treatment) **(B and D)** was assessed using resazurin-based assay. Cells transduced with empty or m*Errfi1*-overexexpression vector (n = 4-5/treatment) were treated with vehicle or CORT (100 nM) for 72 h. In MDA-MB-468 cells, CORT treatment increased **(A)** cell survival (2-way ANOVA; Treatment factor: F(1,8) = 32.11, *P* < 0.0005; Overexpression factor: F(1,8) = 186.8, *P* < 0.0001) and **(B)** viability (2-way ANOVA; Treatment factor: F(1,8) = 16.53, *P* = 0.0012; Overexpression factor: F(1,8) = 38.51, *P* < 0.0001). Overexpression of m*Errfi1* led to marked decrease in cell survival and viability. In MDA-MB-231 cells, CORT treatment significantly enhanced **(C)** colony formation (2-way ANOVA; Treatment factor: F(1,8) = 312.4, *P* < 0.0001; Overexpression factor: F(1,8) = 1.081, *P* = 0.3289) and **(D)** cell viability (2-way ANOVA; Treatment factor: F(1,16) = 14.53, *P* = 0.0015; Overexpression factor: F(1,16) = 0.1916, *P* = 0.6675). Overexpression of m*Errfi1* did not alter these cellular behaviors. For cell viability assay, measures were normalized to raw fluorescence reads at 0 h, which was set as baseline (relative fluorescence units). Normalized values were log_10_ transformed before statistical analysis. Bars represent mean ± standard error of the mean with statistical significance determined through Student’s *t-*test (*P < 0.05, **P < 0.01, ***P < 0.001, ****P < 0.0001 for statistically significant effects of CORT within one expression vector, and ^#^*P* < 0.01, ^##^*P* < 0.001 for statistically significant effects of *ERRFI1* overexpression between the same hormone treatment). Experiments were performed at least twice with consistent results and graphs shown are representative of the different trials.

To complement our knockdown experiments, we overexpressed the mouse homolog of the *ERRFI1* gene in MDA-MB-468 and MDA-MB-231 cells via lentiviral transduction (Supplemental Fig. 6) (59). Stable overexpression of *Errfi1* was not accomplished in MCF10A because the cells failed to proliferate after transduction. In MDA-MB-468 cells, *ERRFI1* overexpression decreased colony formation (Fig. 6A) and cell viability (Fig. 6B) in comparison to empty vector-transduced cells. In MDA-MB-231 empty vector control cells, CORT treatment increased colony formation and this effect of CORT was not affected by *Errfi1* overexpression (Fig. 6C). Similarly, CORT treatment also led to a small increase in the viability of empty vector-transduced lines, and this effect was maintained with *Errfi1* overexpression in MDA-MB-231 cells (Fig. 6D).

## Discussion

GCs, acting via the GR, have been shown to control ductal epithelial cell proliferation and lobuloalveolar morphogenesis during mammary gland development (64,65). Mounting evidence demonstrate that apart from their role in normal mammary epithelia, GCs also contribute to BCa progression (66). A clinical study revealed that BCa patients had significantly higher basal plasma cortisol levels relative to control and that patients with metastatic BCa have elevated cortisol levels compared to early-stage BCa patients (67). Moreover, dysregulation in the diurnal cortisol rhythm that leads to a flatter daytime cortisol slope in metastatic BCa patients was significantly correlated with early mortality (68,69). Elevated GR expression has also been associated with significantly shorter relapse-free survival in both untreated and adjuvant chemotherapy-treated ER-negative BCa patients (70). More recent work on BCa cell lines and patient-derived xenografts discovered higher plasma levels of GCs in mice with metastases concomitant with increased GR activity in metastatic sites (66). In addition, RNA-sequencing of MDA-MB-231 and GR-ChIP sequencing of MCF10A-Myc cells have identified that GC-regulated pathways are enriched for genes involved in neoplastic transformation, cell invasion, and EMT (21,70). Glucocorticoid signaling has also been shown to interact with the hypoxia-inducible factor (HIF) pathway to serve as a stress sensor mechanism in TNBC (71). These stress response pathways act in synergy to upregulate the expression of genes, such as the breast tumor kinase Brk (PTK6), that promote survival and chemoresistance of TNBC cells (72,73). The results from these studies implicate the importance of GC signaling in breast carcinogenesis and metastasis and, together with the persistent use of synthetic GCs in BCa therapy, highlight the need to further uncover GC-regulated genes that may contribute to BCa pathology and response to treatment.

*ERRFI1* is a GC-regulated tumor suppressor gene (23,31) that functions as a negative feedback inhibitor of the EGFR pathway (74) and may therefore mediate some of the effects of GCs in TNBC. In mice, *Errfi1* knockout led to impaired apoptosis of mammary cells (75), overproliferation of keratinocytes, and formation of tumors in various organs (76). Despite several lines of evidence demonstrating the GC-dependent induction of *ERRFI1* (23,31), the molecular basis for this regulation has not been established. In this study, we investigated the mechanism behind the GC-dependent expression of *ERRFI1* and determined the consequence of *ERRFI1* knockdown and overexpression on the behavior of normal mammary epithelia and metastatic TNBC upon GC treatment.

### Molecular basis for glucocorticoid regulation of *ERRFI1*

To replicate the early-to-metastatic transition of BCa in vitro, we selected mammary epithelial cell lines with increasing aggressiveness and metastatic potential. MCF10A cells represent nonmalignant mammary epithelial cells that provide insights in cellular and developmental processes in normal or benign conditions (39). Similar to the established effects of GCs on ductal epithelia cells during normal mammary gland development (65), GCs have also been shown to promote cell proliferation in MCF10A (77). In addition, MCF10A has been widely used as model system to investigate GC-mediated effects on transcriptional regulation and cellular processes, with findings that are consistent with observations in normal mammary epithelia (20,23,44). MDA-MB-468, on the other hand, represents a metastatic TNBC cell line with an EGFR amplification highly associated with poor clinical outcome (40-42). Given that ERRFI1 functions a negative feedback inhibitor of EGFR signaling, we presumed that the function of the GC-*ERRFI1* regulatory axis on different cancer hallmarks will be highlighted in this cell line. We used MDA-MB-231 cells as a model of late-stage, highly aggressive BCa. MDA-MB-231 is a highly metastatic TNBC cell line with mesenchymal-like phenotype (43) and has been used in studies that identified GC-mediated protection from apoptosis and chemotherapeutic resistance (44,66,78).

Analysis of CORT-dependent *ERRFI1* expression in cell line models of normal breast epithelia and TNBC showed that CORT caused a rapid and dose-dependent increase in *ERRFI1* mRNA in the normal breast cell line MCF10A. A similar trend was observed in highly metastatic MDA-MB-231 cells, but with a considerably lower fold induction than that in MCF10A. The pattern of CORT inducible expression of *ERRFI1* mRNA was resistant to protein synthesis inhibition and was abolished with RU486 treatment indicating that *ERRFI1* is a direct GR target in MCF10A and MDA-MB-231 cells. Measurement of the *ERRFI1* pre-mRNA levels in MCF10A and MDA-MB-231 cells showed a similar pattern of expression as the mature transcript suggesting that CORT increases the rate of transcription of the *ERRFI1* gene, consistent with *ERRFI1* being an immediate early response gene (23). In the metastatic MDA-MB-468, a small increase in *ERRFI1* mRNA levels was observed only at a dose of 100 nM, and this level of induction did not change over time.

This difference in the CORT-dependent induction of *ERRFI1* expression in TNBC models relative to normal cells suggests that dysregulation of GC-*ERRFI1* regulatory axis may contribute to the loss of beneficial effects of GC therapy in TNBC. The dampening or loss of GC regulation of *ERRFI1* in TNBC cannot be attributed to differences in GR expression (44) but may be due to changes in methylation patterns of enhancer elements associated with GR response (79), altered posttranslational modification of GR (80), and increased expression of microRNAs (miRNAs) that regulate *ERRFI1* (81). For instance, it has been previously demonstrated that *ERRFI1* basal expression is differentially regulated by DNA methylation and histone acetylation in a cell type-specific manner (82). Moreover, *ERRFI1* can be targeted for degradation by miRNA, such as miRNA-148 and miRNA-200, which have been shown to target *ERRFI1* in glioblastomas and bladder cancer, respectively (81,83). The expression of these candidate miRNAs in response to GC and their role in the GC-dependent regulation of *ERRFI1* expression in BCa warrants further investigation.

Using in silico analysis of publicly available ChIP-seq data for GR, RNA Pol II, and H3K27Ac; DNase I hypersensitivity sites; and GeneHancer long-range interactions, we were able to identify the 821-bp EDE located ~21.5 kb downstream from the *ERRFI1* TSS. The EDE contains 2 candidate GREs and showed CORT-dependent transactivation that was abolished upon pre-treatment with RU486 in MCF10A. To rule out the possibility that the weak dose-dependent and loss of time-dependent induction of *ERRFI1* by GC in MDA-MB-468 is due to a loss of functional GR protein, we compared the activity of the EDE and the known GC-responsive enhancer UCE (50) (Supplemental Fig. 2A and B (59)) in enhancer-reporter assays in MDA-MB-468 cells. We show that the EDE and control UCE exhibited GC-responsive transactivation that was abolished upon treatment with the GR-selective agonist RU486 in MCF10A and MDA-MB-468 cells. These data suggest that MDA-MB-468 cells are capable of eliciting a GR-mediated GC response and that the loss of GC-dependent induction of *ERRFI1* in MDA-MB-468 cells cannot be attributed to a loss of functional GR or impaired GC signaling.

The lack of GR binding at the proximal promoter and our identification of the EDE using enhancer chromatin hallmarks are consistent with several studies showing GR predominantly binds to DNAse-I hypersensitive distal enhancers (84-87) with 86% of GR binding located more than 3 kb from the TSS (86). The EDE is associated with marks of open chromatin indicating that the EDE is already primed and pre-accessible for GR binding, which can peak within 5 min of GC exposure (86). Our in silico analysis demonstrates that the EDE interacts with the *ERRFI1* promoter via chromosomal looping. Distal enhancer elements are enriched in chromatin loops that interact with the promoter (61), and GC can increase the interaction frequency allowing for rapid transcriptional kinetics (86). We hypothesize that the EDE is a GR-bound, GC-responsive *cis*-regulatory element that functions in regulating GC-dependent *ERRFI1* transcription. However, we also cannot eliminate the possibility that there may be other distal *cis*-regulatory and *trans*-regulatory elements that may act via long range interactions and contribute to the GC-dependent regulation of *ERRFI1* in the TNBC lines analyzed in this study.

Enhancer-dependent induction of the *ERRFI1* gene is not unique to GR and can also occur in the presence of other stress stimulus such as hypoxia (88). In luminal breast epithelial cells, hypoxic conditions can induce *ERRFI1* expression via a distal enhancer located ~171 kb upstream of the TSS that is responsive to HIF1α, HIF1β, and HIF2α (88). This implies a potential crosstalk between GC signaling and hypoxia in regulating *ERRFI1* gene expression, especially since GCs can regulate the stability and activity of the HIF family of transcription factors (71,89,90). In MDA-MB-231 cells for instance, the distal upstream enhancer responsive to HIFs (91) also exhibits GR localization which may indicate cooperative action of the two transcription factors in inducing *ERRFI1* gene expression (Supplemental Fig. 7) (59). In contrast, the EDE does not seem to display high HIF1α localization, and a motif search using LASAGNA 2.0 did not reveal high-scoring HIF responsive elements.

### Differential roles of ERRFI1 in TNBC progression

To determine the role of the GC-*ERRFI1* axis in TNBC, we generated stable knockdown of *ERRFI1* and overexpression of *Errfi1* mouse homolog in the 3 triple-negative breast epithelial lines for use in functional assays on CORT-mediated effects on cell proliferation, survival, migration, and apoptosis. We found that regardless of malignancy status, CORT facilitates cell proliferation and migration. This is consistent with findings from different in vitro studies that demonstrate the role of GCs in promoting survival and migration of triple-negative breast epithelial cells (20,44,66,78,92).

Our knockdown experiments showed that *ERRFI1* inhibits cell survival and proliferation in normal breast epithelial model MCF10A. We further observed that in MCF10A, ERRFI1 acts as a negative regulator of the pro-tumorigenic effects of CORT based on the enhanced CORT-mediated increase in cell survival and proliferation when *ERRFI1* expression is reduced. These findings suggest that in normal breast epithelia, ERRFI1 acts as a molecular brake to the general pro-oncogenic GC signaling, consistent with the tumor-suppressive function of ERRFI1 in mammary epithelial cells (23,75) and in several other normal cellular contexts (31,76,93). Interestingly, a microarray study on DEX-treated MCF10A cells demonstrated that activated GR restrains EGFR signaling by upregulating negative feedback regulators such as *ERRFI1* and *DUSP1* while strongly inhibiting expression of EGFR auto-stimulatory ligands such as *TGFA*, *NREG*, *EREG*, and *HBEGF* (23). Our results therefore suggest that while GCs inhibit EGFR signaling, GCs may simultaneously orchestrate a transcriptional program that drives pro-oncogenic properties such as enhanced cell proliferation and cell survival. The anti-proliferative function of ERRFI1 may also be facilitated through the activation of pro-apoptotic *c*-ABL kinase during EGF-deprived conditions (75). Given that GCs repress the production of EGFR ligands, the ability of ERRFI1 to favor apoptosis by direct interaction with *c*-ABL further emphasizes the role of ERRFI1 as an intermediary between GR and EGFR signaling networks.

Similar to its role in MCF10A, ERRFI1 also exhibits an anti-tumorigenic function in MDA-MB-468. *ERRFI1* knockdown in MDA-MB-468 increased cell survival and proliferation and promoted cell migration but did not affect DOX-induced cytotoxicity. The tumor suppressive function of ERRFI1 in MDA-MB-468 is further supported by the decrease in cell survival and proliferation when *Errfi1* is overexpressed. The anti-migratory function of ERRFI1 has been observed in cortical neurons, glioblastoma, (94) and normal mammary epithelia (MCF10A) (23). However, contrary to what we observed in MCF10A, the magnitude of the effect of *ERRFI1* knockdown in enhancing the CORT-mediated increase in cell survival, proliferation, and migration was significantly diminished in MDA-MB-468 cells. This implies that the tumor-suppressive function of ERRFI1 can no longer effectively mitigate the pro-tumorigenic effects of CORT in metastatic BCa and may be attributed to the impaired CORT-mediated induction of *ERRFI1* in this cell line. In addition, the characteristic EGFR amplification (40) in MDA-MB-468 (Supplemental Fig. 8A) (59) may dominate over the anti-tumorigenic effect of ERRFI1 and consequently alter the combined effect of *ERRFI1* knockdown and CORT treatment on cell behavior.

In contrast to the observations in MCF10A and MDA-MB-468, knockdown of *ERRFI1* in the highly aggressive line MDA-MB-231 decreased cell survival, proliferation, and migration and enhanced apoptosis indicating that ERRFI1 has a pro-tumorigenic effect. Our results are consistent with the finding that ERRFI1 is required for 3D outgrowth and pulmonary colonization by MDA-MB-231 cells in mice (41). However, *Errfi1* overexpression did not confer pro-tumorigenic effects possibly due to the relatively high basal mRNA levels of *ERRFI1* in this cell line (Supplemental Fig. 8B (59,95)). The observed paradoxical shift in ERRFI1 function in MDA-MB-231 is complemented by the transition of EGFR into an anti-tumorigenic factor in the highly aggressive stages of BCa (41), and the distinct downregulation of EGFR as tumors evolve into metastatic forms (95,96).

The seemingly contradictory role of ERRFI1 in highly aggressive TNBC cells may be explained by its nature as a cytoplasmic adaptor protein and the many factors that influence its transcription (97). While most research on ERRFI1 has focused on its role as an inhibitor of the EGFR pathway, this protein also contains other interaction domains including a Cdc42- and Rac-interactive binding domain, a Src-homology 3-binding moiety, and a 14-3-3 protein-binding motif (27), affording ERRFI1 the flexibility to participate in a variety of signaling pathways. For instance, the direct interaction between ERRFI1 and GRB2 may preclude activation of the mitogen-activated protein kinase pathway and confer tumor suppressive effects (98). On the other hand, the preferential binding of ERRFI1 to the AKT inhibitor PHLLP in cells with low EGFR expression reinforces AKT signaling and may therefore promote cell survival (99). Lastly, ERRFI1 can also orchestrate a pro-apoptotic action in the cell by binding to *c*-ABL in low EGFR activity conditions (75). It is therefore tempting to speculate that the stoichiometry of the ERRFI1 interactome and the status of the GR transcriptional network dictate the overall function of the GC-*ERRFI1* regulatory axis in TNBC.

In summary, our findings present the regulatory basis of the GC-dependent induction of *ERRFI1* and the potential contributions of the GC-*ERRFI1* axis in the TNBC phenotype. To our knowledge, this study is the first to uncover the molecular mechanism for how GCs modulate *ERRFI1* expression. Furthermore, our results from the orthogonal cellular assays collectively demonstrate that GCs act as pro-oncogenic signals in TNBC and that ERRFI1 plays divergent roles in TNBC progression. Consequently, the paradoxical transition of ERRFI1 from a tumor suppressor that can mitigate the oncogenic effect of GC to a tumorigenic factor in advanced TNBC, in addition to the complex transcriptional network coordinated by GR, may partially explain the ineffective or adverse outcomes of GC therapy in TNBC.

## Additional Information

***Disclosure Summary:*** The authors have nothing to disclose.

***Data Availability.*** All data generated or analyzed during this study are included in this published article or in the data repositories listed in References.
